# *CADEM*: calculate X-ray diffraction of epitaxial multilayers

**DOI:** 10.1107/S1600576716018379

**Published:** 2017-02-01

**Authors:** Paulina Komar, Gerhard Jakob

**Affiliations:** a Institute of Physics, University of Mainz, Staudinger Weg 7, 55128 Mainz, Germany; b Graduate School Materials Science in Mainz, Staudinger Weg 9, 55128 Mainz, Germany

**Keywords:** superlattices, epitaxial multilayers, X-ray diffraction pattern calculation, computer programs

## Abstract

Epitaxial multilayers and superlattice (SL) structures are gaining increasing importance as they offer the opportunity to create artificial crystals with new functionalities. These crystals deviate from the parent bulk compounds not only in terms of the lattice constants but also in the symmetry classification, which renders calculation of their X-ray diffraction (XRD) patterns tedious. Nevertheless, XRD is essential to get information on the multilayer/SL structure such as, for example, out-of-plane lattice constants, strain relaxation and period length of the crystalline SL. This article presents a powerful yet simple program, based on the general one-dimensional kinematic X-ray diffraction theory, which calculates the XRD patterns of tailor-made multilayers and thus enables quantitative comparison of measured and calculated XRD data. As the multilayers are constructed layer by layer, the final material stack can be entirely arbitrary. Moreover, *CADEM* is very flexible and can be straightforwardly adapted to any material system. The source code of *CADEM* is available as supporting material for this article.

## Abstract 2

This article presents a powerful yet simple program, based on the general one-dimensional kinematic X-ray diffraction (XRD) theory, which calculates the XRD patterns of tailor-made multilayers and thus enables quantitative comparison of measured and calculated XRD data. As the multilayers are constructed layer by layer, the final material stack can be entirely arbitrary.

## 1. Introduction and motivation   

With the increasing availability and capability of layer-by-layer deposition techniques, epitaxial thin films and superlattices (SLs) consisting of different materials are now widespread in scientific research. Further growth in this field can be foreseen, as in such epitaxial multilayers new effects are observed that are not present in the parent compound; the two-dimensional electron gas at the LaAlO_3_/SrTiO_3_ interface (Stephanovich *et al.*, 2016 ▸; Pesquera *et al.*, 2014 ▸; Annadi *et al.*, 2013 ▸), ferroelectricity in PbTiO_3_/SrTiO_3_ (Zubko *et al.*, 2016 ▸; Dawber *et al.*, 2007 ▸) or SrTiO_3_/BaTiO_3_/CaTiO_3_ SLs (Lee *et al.*, 2005 ▸), and a topological insulator state in strained HgTe heterostructures (Brüne *et al.*, 2011 ▸) are a few examples. The first and one of the most important characterization methods of such epitaxial structures is X-ray diffraction (XRD), as the peak positions encode the lattice periodicities. However, more information can be gained by evaluation of the full diffraction pattern including the peak shapes. While numerous powerful program codes that calculate the XRD patterns of single crystals already exist, their practical use is limited as they are not adapted to the specific problem. For example, for SLs consisting of two materials *A* and *B* with a stacking of two unit cells of each material (*A*_2_ *B*_2_)_*n*_ repeated *n* times, one has to create a new crystallographic cell (*AB*) which belongs to a specific point group as input for the program. Changing only the layering sequence, for example to (*A*_3_ *B*_5_)*_n_*, usually requires one to select a new point group, starting the input all over again. In addition, most often the fact that *n* is limited is not accounted for, nor is the possibility of asymmetric strain relaxation at the substrate interface or at the interface between the two components. As the resulting point group symmetry is low and the unit cell is large, a high number of reflections will be calculated where the majority are unobservable in a standard Bragg–Brentano geometry.

For practical purposes it is therefore more convenient to use an adapted program code that calculates only the observable reflections of the SL. Here dedicated programs for different material classes exist. For example,  *SUPREX* (Fullerton *et al.*, 1992 ▸) does the job for high-*T*  superconductor SLs. However, the program is written in Fortran (and another version exists in Turbo Pascal) and extension to different material classes is non-trivial. For metallic type SLs, calculations based on realistic sample structures using Monte Carlo methods were demonstrated to reproduce nicely experimental diffraction patterns and could simulate also the low-angle diffraction data where a dynamical calculation is needed (Gładyszewski, 1989 ▸, 1991 ▸). Yet these codes did not find very widespread application irrespective of their power, as the degree of complexity of the modeling is proportional to the complexity of the computer code. In this article we discuss a minimal program designed to ‘calculate X-ray diffraction patterns of epitaxial multilayers’ (*CADEM*), thin films, SLs and nonperiodic structures. We describe all details of the calculation explicitly in the supporting material in order to enable further extensions of the program code by others. Below, on the other hand, we demonstrate three examples of the application of *CADEM*.

Because we use a high-level language (MATLAB; The MathWorks Inc., Natick, MA, USA), the code is extremely compact and enables adaptation to other material systems or porting to other languages such as, for example, Python, C, C++ *etc.* We believe that anybody who has some experience in programming in general will be able to translate the code to the chosen programming language as the only MATLAB-specific operations are standard matrix operations, *i.e.* vector × vector or matrix × vector, for which corresponding routines are available. The program can run from the command line with parameter changes made in the input file *via* a text editor, and modification of this computational core of the code should not cause issues. Any alteration of the graphical user interface is more demanding and requires deeper knowledge of MATLAB and the corresponding porting language.

The correct operation of *CADEM* was verified on examples of half-Heusler materials and vanadium. However, one can implement any material system. An example based on SrTiO_3_ and SrRuO_3_ perovskites is provided. The program was tested on the following versions of MATLAB: R2012a, R2012b, R2013a, R2014a, R2014b, R2015a and R2015b. No additional toolboxes are required.

Nevertheless, care must be taken in interpretation of the results as X-ray peak intensities in general cannot be uniquely converted to real-space structures owing to loss of the phase information. Thus physical intuition is needed for selection of the possible real-space structures. For verification, one can use transmission electron microscopy (TEM) to examine the cross section of the film. However, the TEM analysis of thin films is destructive and time consuming. On the other hand, XRD is easily accessible, simple to perform, quick and does not require any additional sample preparation steps. As shown by Komar *et al.* (2016 ▸), both the XRD fitting procedure presented in detail here and the analysis of TEM images give on average the same result regarding the SL period. We also emphasize that TEM provides very localized information, whereas XRD allows one to obtain the average SL period from a much larger volume of the sample.

**Figure 1:**
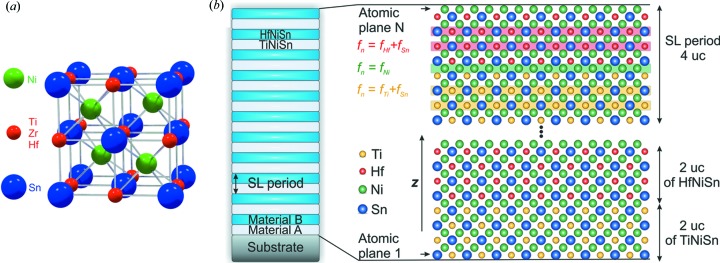
(*a*) The unit cell of the half-Heusler *M*NiSn materials, where *M* = Ti, Zr, Hf. (*b*) A schematic representation of the TiNiSn/HfNiSn SL.

The main motivation for this work was research focused on half-Heusler (HH) SLs. Our approach presented in detail here has already been successfully employed in several contributions (Komar *et al.*, 2016 ▸; Hołuj *et al.*, 2015 ▸; Jaeger *et al.*, 2014 ▸). HH materials have a general formula *M*NiSn (*M* = Ti, Zr, Hf) and crystallize in the MgAgAs structure (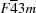, space group No. 216) (Jeitschko, 1970 ▸). Fig. 1 ▸(*a*) shows the arrangement of the atoms in the unit cell (uc). The lattice constants are equal to 5.941, 6.113 and 6.083 Å for TiNiSn, ZrNiSn and HfNiSn, respectively (Jeitschko, 1970 ▸). As shown by Komar *et al.* (2016 ▸) and Jaeger *et al.* (2011 ▸), the HH compounds grow epitaxially on top of MgO(001) with 45° in-plane rotation.

## 2. Calculation details   

To calculate the intensity of diffracted X-rays we used one-dimensional kinematic diffraction theory, discussed in detail in the supporting material. Prior to the calculation we construct the material atomic plane by atomic plane along the **z** direction, as shown in Fig. 1 ▸(*b*). Because the unit cell of HH materials may be divided into four atomic layers, forming an *M*Sn/Ni/*M*Sn/Ni stack, the calculation accuracy is not limited to a single uc, but to a quarter of the uc instead. Thus, we build a material that has a thickness equal to *N* atomic planes, *i.e.* 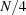
 uc, with alternating *M*Sn and Ni layers (substrate/*M*Sn/Ni/*M*Sn/Ni/…/*M*Sn/Ni/air). The position of the first atomic plane is determined by the out-of-plane lattice constant of the HH material, 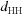, namely 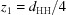, and the position of the *N*th plane corresponds to the total film thickness 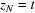.

The stack shown in Fig. 1 ▸(*b*) is composed of three kinds of atomic layers, highlighted in red, green and yellow. Each of these atomic planes scatters X-rays differently, and therefore atomic scattering factors were assigned to each layer, depending on the composition. Consequently, we have 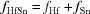 for HfSn layers (red), 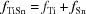 for TiSn layers (yellow) and 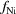 for Ni layers (green).

For more details we refer to the supporting material, where one can find a discussion of the theoretical background and a way to achieve realistic peak shapes by convolution of the diffraction peaks based on measured empirical parameters.

## 3. Application examples   

### 3.1. The influence of disorder   

**Figure 2:**
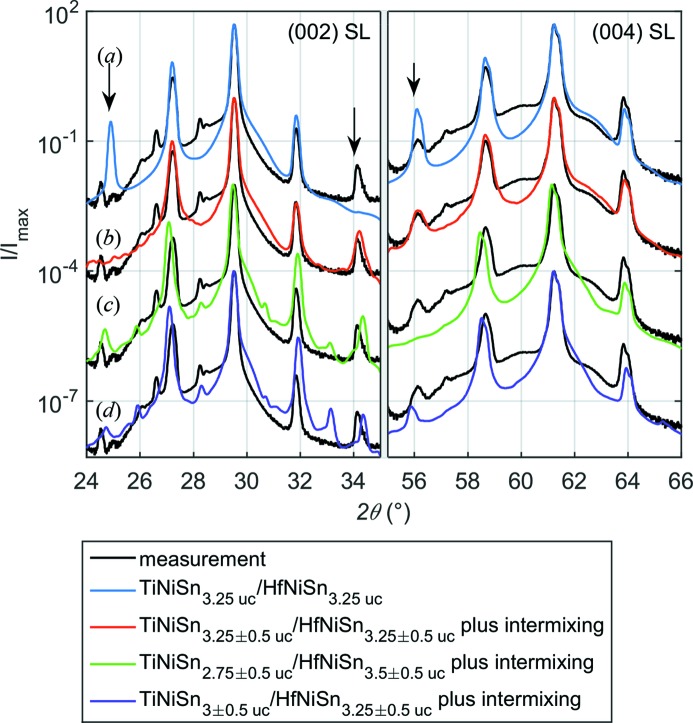
Measured (black) and calculated (colored lines) 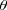
–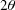 XRD patterns of (*a*) the perfect [(TiNiSn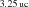/HfNiSn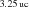) × 200, blue] and (*b*) the distorted [(TiNiSn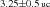/HfNiSn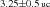) × 200, red] SLs. The arrows indicate the reflections that exhibit the greatest differences in the intensity between the measurement and the calculation with constant SL period. (*c*), (*d*) Two examples of patterns that reproduce the weak peak at 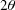 = 28.3° but do not coincide with the positions of more intense satellites. Curves are shifted vertically for clarity.

For relatively thick SLs, having an SL period greater than ∼10 nm, one can successfully determine the SL period and the lattice parameters of both materials independently. On the other hand, for smaller periods it is possible to fit only the SL period and the mean lattice spacing. This is because the peaks corresponding to TiNiSn and HfNiSn are no longer separated, and their XRD patterns exhibit one main diffraction peak instead (see Fig. 2 ▸). Moreover, thinner SL periods cause greater separation between the satellite peaks as in the case of the (TiNiSn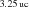/HfNiSn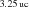) × 200 SL presented in Fig. 2 ▸(*a*). Therefore, for short-period SLs it is easier to notice some remaining discrepancies between the measured and calculated intensities of the satellite peaks. The arrows in Fig. 2 ▸(*a*) indicate the reflections that exhibit the greatest differences in the intensity between the calculation and the measurement. Substituting 50% of TiNiSn (HfNiSn) atoms at the interface for HfNiSn (TiNiSn), and replacing the constant SL period with its Gaussian distribution (TiNiSn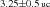/HfNiSn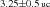) × 200 we are able to obtain a much better agreement (see Fig. 2 ▸ *b*). Being more specific, the intermixing can be achieved by the modification of the atomic scattering factors and the lattice constants at the interfaces. Namely, if the last layer of TiNiSn is TiSn, 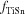 becomes 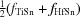. The atomic scattering factor for Ni, *i.e.* 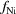, stays unchanged. Moreover, the out-of-plane lattice constants of these two layers at the interface become equal to 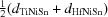.

Although patterns (*a*) and (*b*) fit the experimental data well, some features, such as a weak reflection at 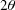 = 28.3°, are still not reproduced. This feature appears when the difference between the mean thicknesses of constituent layers is equal to 0.25 uc × 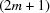, where *m* is an integer, as illustrated in Figs. 2 ▸(*c*) and 2 ▸(*d*). In both cases the peak appears. However, the positions of other satellites do not coincide with the measured reflections anymore. To solve this problem it would be necessary to consider several grains with slightly different periodicities and sum the intensities of diffracted rays, *i.e.* treating the scattering from different grains incoherently. The current version of *CADEM* takes into account disorder only in a coherent fashion within a single grain, for example, by a Gaussian distribution (or any arbitrary set) of layer thicknesses within a single grain, and sums the scattering factors.

### 3.2. XRD patterns of aperiodic multilayers   

**Figure 3:**
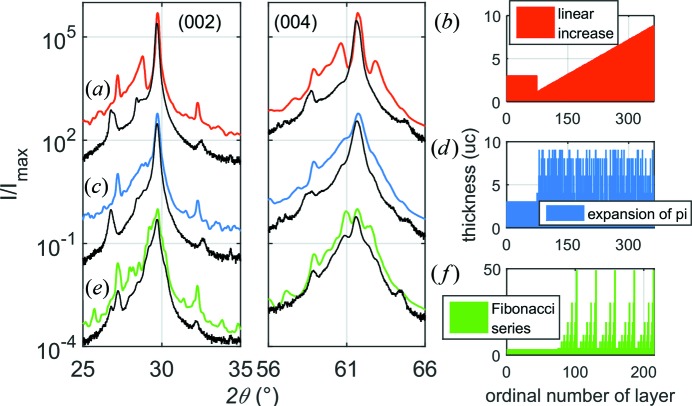
(*a*), (*c*), (*e*) XRD patterns of the layer designs shown in (*b*), (*d*) and (*f*), respectively. Black data represent measured patterns for respective layer stacks. Curves are shifted vertically for clarity.

Our approach, based on the layer-by-layer structuring of the film, provides enormous flexibility in defining the arrangement of layers in multilayer structures. Thus, we are not limited to the investigation of SLs with a fixed period, but *CADEM* can simulate multilayers having an arbitrary structure. As an example, in Fig. 3 ▸ we present the calculated patterns of three non-periodic ∼1 µm-thick TiNiSn/HfNiSn layer designs. We calculated a structure (*a*), (*b*) with linearly increasing layer thicknesses (topmost, red), (*c*), (*d*) having random thicknesses identical to the expansion of π number (*i.e.* 3 uc TiNiSn/1 uc HfNiSn/4 uc TiNiSn/1 uc HfNiSn/5 uc TiNiSn/9 uc HfNiSn/…) (central, blue), and (*e*), (*f*) with layer thicknesses based on the Fibonacci series (bottommost, green) on top of a periodic SL. From these three datasets one can notice that every XRD pattern has its own characteristic features, which distinguish it from the others. Moreover, the measured patterns (black data) exhibit a high degree of similarity compared to the calculated ones (colored lines), especially in the case of the Fibonacci series. That makes *CADEM* a versatile and valuable tool. However, while the program is able to predict any possible change in the diffraction patterns caused by variations of the layering sequence, an inversion is not possible. Nevertheless, it can be used for quality checking of the prepared structures.

### 3.3. Strain and asymmetric Laue oscillations   

Whenever the X-ray coherence length is at least equal to the film thickness and the film is smooth enough, the XRD patterns exhibit Laue oscillations (Ying *et al.*, 2009 ▸). Here we present these numerous oscillations *via* an example of DC sputtered vanadium, grown on MgO(001). The intensities of the satellite peaks are not symmetric with respect to the main diffraction peak. Such an asymmetry is evidence of strain normal to the film (Robinson & Vartanyants, 2001 ▸; Vartanyants *et al.*, 2000 ▸). In order to introduce the strain into the film we modified the spacing between adjacent atomic layers using exponential [equation (1)] and power law [equation (2)] relaxation:
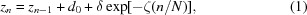


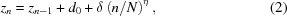
where 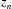 denotes the position of the atomic layer with respect to the previous one, 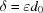 is expressed as a product of the relative strain ∊ and the distance between atomic planes 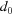 in the non-strained case, η is the exponent of the power law, and ζ is the numeric factor of the exponent. The relative strain is defined as 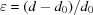, where *d* is the strained interplanar spacing. The strain relaxation models can be found in the file CADEM.m. To apply them one has to uncomment the required model.

**Figure 4:**
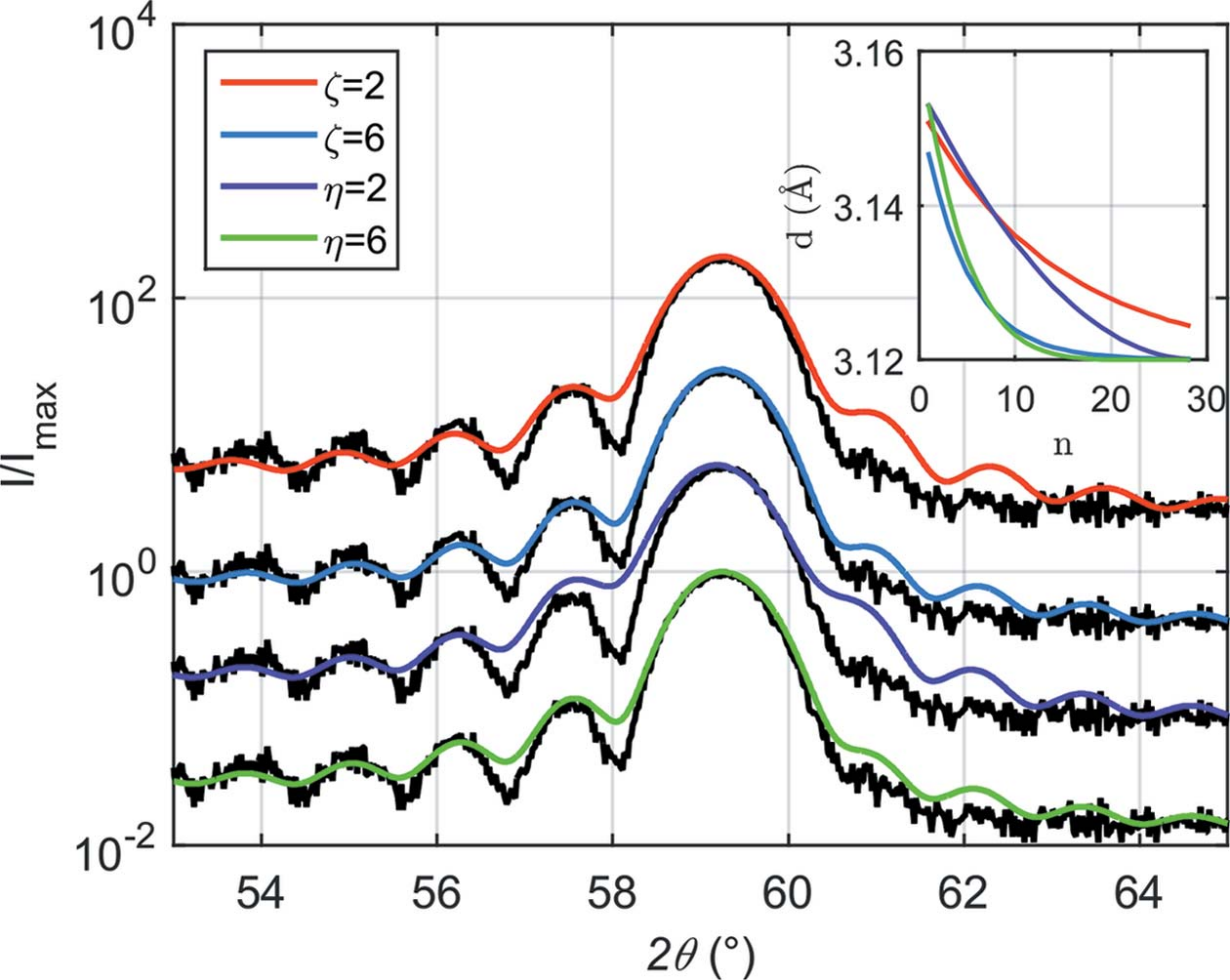
Measured (black data) and calculated (smooth solid lines) patterns of a vanadium thin film (8.4 nm). The calculation was performed using the variable strain relaxation models that are specified in the legend. Curves are shifted vertically for clarity. Inset: strained interplanar spacing (*d*) *versus* the ordinal number of the unit cell counting from the substrate (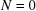) towards the air (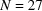).

As shown in Fig. 4 ▸, the higher the value of both η and ζ, the greater the suppression of the high-angle satellites becomes. For η and ζ equal to 6 we achieved very good resemblance between the measured and calculated patterns. This indicates that strain relaxes quickly close to the substrate interface, while a coherent uniform strain persists as the lattice constant for the film is still different from the bulk. As demonstrated in the inset in Fig. 4 ▸, the distribution of lattice constants as a function of distance from the substrate–film interface is essentially identical in the case of 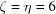. Therefore, both of the models are appropriate to study strain relaxation mechanisms.

Remarkably the thickness of 8.4 nm determined from the Laue oscillations is 2.1 nm smaller than that determined from low-angle reflectometry data (not shown). This is a correct result as the top surface is oxidized and the electron density of the vanadium oxide will be similar to that of the metal, so both contribute to the dynamical scattering at low angles. However, the (presumably amorphous) oxide layer will not contribute to the coherent scattering from the metallic epitaxial planes.

## 4. Summary   

The powerful yet simple approach presented in detail in this article with the example of the HH SLs is based on the general one-dimensional kinematic X-ray diffraction theory. The great flexibility of *CADEM* concerns not only the possibility to generate XRD patterns of arbitrarily defined layer sequences, but also the fact that one can adapt it to any compound, just by changing the material-dependent parameters summarized in part IIIC of the supporting material and in the file constants.m. Moreover, it is important to stress that the shape of the reflections was modeled on the basis of the empirically obtained parameters. Therefore, one can easily modify it to the specific requirements in order to get reasonable qualitative estimations.

We have demonstrated three possibilities of using this program: (1) determination of the SL period and the out-of-plane lattice constants in periodic SLs and estimations of intermixing, (2) generation of the XRD patterns from arbitrarily defined multilayers, and (3) determination of the film thickness and the mechanism of strain relaxation from the Laue oscillations in a thin film.

We encourage readers to download the supporting material with the source code of *CADEM* and explore its functionality by running the file run_CADEM_GUI.m.

## 5. Related literature   

For further literature related to the supporting information, see Buerger & Klein (1945 ▸), MacGillavry & Rieck (1968 ▸), Ibers & Hamilton (1974 ▸), Dauben & Templeton (1955 ▸), Peng *et al.* (1996 ▸), Birkholz *et al.* (2006 ▸) and Azároff (1955 ▸).

## Supplementary Material

Supporting information. DOI: 10.1107/S1600576716018379/vh5063sup1.pdf

The source code of CADEM. DOI: 10.1107/S1600576716018379/vh5063sup2.zip
