# Supplementary material for: CADEM: calculate X-ray diffraction of epitaxial multilayers
Source: J Appl Crystallogr. 2017 Feb 1;50(Pt 1):288–92. doi: 10.1107/S1600576716018379 (PMC5294394; doi:10.1107/S1600576716018379)
Supplement: Supplementary file 1 [file j-50-00288-sup1.pdf]

CADEM - CAlculate X-ray Diffraction of Epitaxial Multilayers Supplementary Material

Paulina Komar[1, 2, *] and Gerhard Jakob[1, 2]

[1] Institute of Physics, University of Mainz, Staudinger Weg 7, 55128 Mainz, Germany.
[2] Graduate School Materials Science in Mainz, Staudinger Weg 9, 55128 Mainz, Germany.

(Dated: October 31, 2016)

I. THEORETICAL BACKGROUND

Due to the well-defined orientation of epitaxial films the XRD patterns exhibit only very specific reflections in the standard Bragg-Brentano geometry of x-ray diffractometers. Namely, $\theta - 2\theta$ XRD patterns contain only the reflections of the crystallographic lattice planes that are perpendicular to the surface normal. Thus, in the case of the HH materials presented here, only (002) and (004) film reflections are visible at the angles θ defined by Bragg's law

$$n\,\lambda = 2\,d\,\sin\theta, \tag{1}$$

where θ is an incident angle, λ is the radiation wavelength, n is an integer labeling the order of the peak, and d is the distance between the atomic planes. If, on the other hand, instead of a single film, a SL is considered, additional reflections, called satellite peaks, appear around the main diffraction peaks at the angles satisfying equation

$$\frac{2\,\sin\theta}{\lambda} = \frac{1}{\bar{d}} \pm \frac{n}{\Lambda}. \tag{2}$$

In this case, $\bar{d}$ is the average distance between the atomic planes, taking into account both materials, n is the order of the satellite peak, $\Lambda = \bar{d}\cdot(N_A + N_B)$ is the SL period, and N_A (N_B) is the number of atomic planes of material A (B) in one SL period.

To calculate the intensity of diffracted x-rays we used one dimensional kinematic diffraction theory where I is represented by [1]

$$I = L\cdot p\cdot A\cdot F^2. \tag{3}$$

The symbol L stands for the Lorentz factor, p is the polarization factor, A is the absorption factor, and F is the structure factor.

As the x-rays are not strictly monochromatic and diverge a little, constructive interference of the diffracted rays takes place not only at the predicted Bragg angle, but also in its vicinity. The Lorentz factor [1]

$$L = \frac{1}{\sin 2\theta} \tag{4}$$

accounts for this effect.

Depending on the direction of polarization of the incident wave, a result of diffraction can be altered. The change is maximal if the polarization of x-ray is in the plane of scattering. If, on the other hand, the polarization is perpendicular to the plane of scattering, the amplitude of an electromagnetic wave is not affected. Therefore, for unpolarized waves leaving the x-ray tube, p takes a form of Eq. (5a) [1, 6, 7]. In case when the beam passes through a monochromator (e.g. Ge monochromator, or Göbel mirror), the beam used for the diffraction experiment is not unpolarized any more. Thus, the Bragg angle of the monochromator (θ_M) must be included in the polarization factor, as shown in Eq. (5b) [7]. The factor p is rather insignificant ($\frac{1}{2} \leqslant p \leqslant 1$) and it depends on the optical elements used during the experiment. By default it is not taken into account in our calculations, however, one can enable it by uncommenting the respective lines in the code in *CADEM.m*.

$$p = \frac{1+\cos^2 2\theta}{2} \tag{5a}$$

$$p = \frac{1+\cos^2 2\theta\cos^2 2\theta_M}{1+\cos^2 2\theta_M} \tag{5b}$$

According to Eq. (3), the scattered intensity scales with the square of the structure factor F, which is given by

$$F(\boldsymbol{q}) = \sum_{n=1}^{N} f_n(\boldsymbol{q})e^{i\Delta\boldsymbol{q}\boldsymbol{z_n}}. \tag{6}$$

The coefficient N is the total number of the atomic planes taken for the XRD calculation, $\boldsymbol{z_n}$ is the position of an atomic plane, $\Delta\boldsymbol{q} = \boldsymbol{k'} - \boldsymbol{k}$ is a momentum transfer that has the absolute value $|\Delta\boldsymbol{q}| = \frac{4\pi\sin\theta}{\lambda}$, and $f_n(\boldsymbol{q})$ is an angle dependent scattering factor that can be well approximated using an analytical expression [3]

$$f_n\left(\frac{\sin\theta}{\lambda}\right) = \sum_{i=1}^{4} a_i e^{-b_i\left(\frac{\sin\theta}{\lambda}\right)^2} + c. \tag{7}$$

The factors a_i, b_i, and c are the element dependent coefficients tabulated in the International Tables for X-ray Crystallography [3] and also summarized in the Table I for HH materials and vanadium.

In the calculation of the atomic scattering factor, according to Eq. (7), it is assumed that the binding energies

* holuj@uni-mainz.de, komarpaulina@outlook.com

TABLE I. The numerical factors used to calculate the atomic scattering factors and the linear attenuation coefficients for vanadium and half-Heusler MNiSn materials, where $M = (\mathrm{Ti, Zr, Hf})$ [2–5].

	**Ti**	**Ni**	**Zr**	**Sn**	**Hf**	**V**
a_1	9.760	12.838	17.877	19.189	29.144	10.297
a_2	7.356	7.292	10.948	19.101	15.173	7.351
a_3	1.699	4.444	5.417	4.459	14.759	2.070
a_4	1.902	2.380	3.657	2.466	4.300	2.057
b_1	7.851	3.879	1.276	5.830	1.833	6.866
b_2	0.500	0.257	11.916	0.503	9.600	0.439
b_3	35.664	12.176	0.118	26.891	0.275	26.893
b_4	116.105	66.342	87.663	83.957	72.029	102.478
c	1.281	1.034	2.069	4.782	8.582	0.586
$\Delta f'$	0.2	-3.1	-0.6	-0.7	-6.0	0.2
$\Delta f''$	1.9	0.6	2.5	5.8	5.0	2.3
B (Å^2)	0.5261	0.3644	0.5822	1.1596	0.4100	0.5855
μ/ρ | Cu K$_\alpha$	202.4	48.8	136.8	253.3	157.7	222.6
(cm^2/g) | Cu K$_\beta$	153.2	282.8	101.3	193.1	121.0	168.0

of all electrons in an atom are small compared to the energy of the incident x-ray photon. In case when the atom has an absorption edge not far from the incident x-ray energy, the dispersion corrections should be taken into account. Then, the atomic scattering factor becomes

$$f = f_n + \Delta f' + i\Delta f''. \tag{8}$$

The dispersion correction was applied to f of the elements forming the HH materials due to relatively small difference between the Cu $K_{\alpha 1}$ radiation wavelength 1.541 Å and the Ni K absorption edge that corresponds to the wavelength 1.488 Å, and is observed at 8.331 keV [2]. The correction factors can be found in the International Tables for X-ray Crystallography [2–4] and are summarized in Table I.

The final amendment, which has to be taken into account, corresponds to the fact that in real systems the atoms are not stationary, but they vibrate around their average positions. That results in a decrease of the peak intensity and an increase of the background intensity. This temperature dependent effect is described by the Debye-Waller factor $e^{-B\left(\frac{\sin\theta}{\lambda}\right)^2}$. The atom dependent constants B are summarized in Table I [5]. Taking into account all the effects listed above, the total scattering factor has the form

$$f_n\left(\frac{\sin\theta}{\lambda}\right)=\left(\left(\sum_{i=1}^{4} a_i e^{-b_i\left(\frac{\sin\theta}{\lambda}\right)^2}+c\right)+\Delta f'+i\Delta f''\right)e^{-B\left(\frac{\sin\theta}{\lambda}\right)^2}. \tag{9}$$

In case when the diffracting planes are composed of more than one element, f is calculated as a sum. Assuming that in a plane AB_2 there is one atom A and two atoms B, the atomic scattering factor is equal to $f_{AB_2} = f_\mathrm{A} + 2 \cdot f_\mathrm{B}$.

A factor not discussed yet, is the x-ray absorption in matter. It is known that the x-ray intensity I_0 that enters any material will be attenuated to an amount $I_0 e^{-\mu t}$ after passing a thickness t. The parameter μ is the linear attenuation coefficient that depends on the radiation wavelength and chemical composition of the investigated sample. One may be tempted to break this absorption down to an absorption in every atomic layer and include this in Eq. (6). However, this is in conflict with the quantum mechanical nature of the scattering process and the preconditions of the kinematic theory. In essence an x-ray photon sees a coherent scattering volume that is either limited by its coherence length or the coherence of the atomic planes of the crystallite. During one scattering event all atoms interact with the photon with equal strength. However, if the beam penetrates further, the absorption needs to be included with its corresponding angle dependent path length.

Considering the Bragg-Brentano configuration, the absorption factor is equal to [6]

$$A = 1 - e^{-\frac{2\mu t}{\sin\theta}}. \tag{10}$$

The mass attenuation coefficients μ/ρ (ρ is the density of a material) are available in the International Tables for X-ray Crystallography [3] and are summarized in Table I for both Cu K$_\alpha$ and Cu K$_\beta$ radiation lines.

The linear attenuation coefficients for MNiSn are calculated by applying the mass attenuation coefficients μ_m for every element of the compound, i.e. M=(Ti or Hf), Ni, and Sn, the atomic masses m of respective elements,

mass $m_{M\mathrm{NiSn}}$, and the density $\rho_{M\mathrm{NiSn}}$ of the compounds according to

$$\mu_{M\mathrm{NiSn}} = \frac{\mu_{m,\,M} \cdot m_M + \mu_{m,\,\mathrm{Ni}} \cdot m_{\mathrm{Ni}} + \mu_{m,\,\mathrm{Sn}} \cdot m_{\mathrm{Sn}}}{m_{M\mathrm{NiSn}}} \cdot \rho_{M\mathrm{NiSn}}. \quad (11)$$

The total linear attenuation coefficient is determined based on an effective amount of both compounds that form the SL structure. Namely, if the total amount of TiNiSn (HfNiSn) is equal to the thickness t_A (t_B), μ is represented by the effective attenuation coefficient

$$\mu_{\mathrm{TiNiSn/HfNiSn}} = \frac{\mu_{\mathrm{TiNiSn}} \cdot t_A + \mu_{\mathrm{HfNiSn}} \cdot t_B}{t_A + t_B}. \quad (12)$$

II. MODELING OF DIFFRACTION PEAKS

A simple calculation of the intensity of diffracted x-rays, using solely Eq. (3), is not sufficient to fully model the diffraction peaks. As demonstrated in Fig. 1 a, the calculation (red) of 1 μm thick TiNiSn film reproduces well the peak position, however, the shape of the calculated peak completely differs from the measured one. The full width at half maximum (FWHM) of the calculated reflections is $\sim 0.01°$, whereas the measured peak width is $\sim 0.17°$. Such a broadening is caused by both the experimental setup and the sample itself. The instrumental contribution arises from factors such as the finite physical size of the radiation source, axial divergence of the incident or diffracted beams, and any misalignment of the diffractometer, among others. The contribution to the peak broadening coming from the sample is caused by, for example, the finite size of the diffracting domains/crystallites, crystal lattice distortion caused by any point and/or line defects, and strain.

Next to the extremely sharp diffraction peak visible in Fig. 1 a, the calculated pattern exhibits also many orders of oscillations. These numerous oscillations are called the Laue oscillations, or the thickness fringes, and are created when the x-rays are diffracted coherently from the total film thickness, i.e. if the x-ray coherence length is at least equal to the film thickness [8]. In real 1 μm thick TiNiSn films the crystallite size is much smaller ($\sim$ 100 nm), therefore, the Laue oscillations are suppressed and the peak is broadened (see black data in Fig. 1 a). One can in principle model both the instrumental peak broadening and the crystallite size broadening in the computer to achieve a realistic peak shape as was demonstrated by Gładyszewski using a Monte-Carlo method to simulate scattering of SLs consisting of finite sized grains [9, 10]. Here we restrict our calculation to a more simple empirical peak broadening. As discussed below the measured peak shape of a suitable 'parent' compound provides a simple but suitable starting point in order to estimate peak broadening caused by the heterostructure effects.

As reference peaks for the simulation of the HH heterostructures we used the (002) and (004) peaks of thick TiNiSn films measured with identical machine settings. The increased angular resolution at higher 2θ angles in Bragg-Brentano geometry leads to a different peak shape at higher angles. However, all SL peaks occur close to those reference peaks. Already a convolution of the calculated data with a Gaussian singlet provides a good resemblance to the measured pattern, as summarized in Fig. 1 b and d (blue curves). However, the usage of the singlet, having an arbitrary value $\sigma = 0.1°$ for both (002) and (004) film reflections, does not reproduce the observed peak asymmetry arising from the presence of Cu $K_{\alpha 1}$ (1.54051 Å) and Cu $K_{\alpha 2}$ (1.54433 Å) radiation wavelengths.

Clearly a peak doublet (due to Cu $K_{\alpha 1}$ and Cu $K_{\alpha 2}$), or even more precisely a peak triplet (due to Cu $K_{\alpha 1}$, Cu $K_{\alpha 2}$, and Cu K_{β}), is needed to model the peak shape accurately. Even though the intensity of the Cu K_{β} line is relatively low, it is clearly visible in the logarithmic scale, which is frequently employed to visualize the satellite peaks. To avoid arbitrariness in the choice of the peak shape and its parameters, the triplets of four different peak shapes (Gaussian, Lorentzian, Pseudo-Voigt, and Pearson VII) were fitted to the XRD data measured for 1 μm thick TiNiSn film. The comparison of the performed fits for the (004) reflection is summarized in Fig. 2. Based on the coefficient of determination, R^2, we can deduce that the best fits were achieved for the Pseudo-Voigt and Pearson VII line shapes, with a slight advantage in favor of the Pseudo-Voigt. Therefore, for the further calculations we used the Pseudo-Voigt fitting parameters. As the separation between these three reflections increases with the 2θ angle, the (002) and (004) diffraction peaks were fitted separately.

The resulting calculation for TiNiSn film is demonstrated in Fig. 1 c, whereas Fig. 1 e presents the patterns for ($\mathrm{TiNiSn}_{21.5\,\mathrm{uc}}$/$\mathrm{HfNiSn}_{21.5\,\mathrm{uc}}$)$\times$29 SL (both patterns are green). Based on these computed patterns, one can precisely determine any change of the out-of-plane lattice constants in respect to the bulk values by fitting the position of the main diffraction peak. Moreover, fitting the period of the satellite peaks one can easily obtain the information about the exact thicknesses of both layers that form the SL period.

Having a more general look on these patterns, one can notice a substantial improvement of the fit, compared to the first type of the convolution. Here, not only the peak positions, but also the overall peak shape becomes more similar to the measured. Moreover, to show that

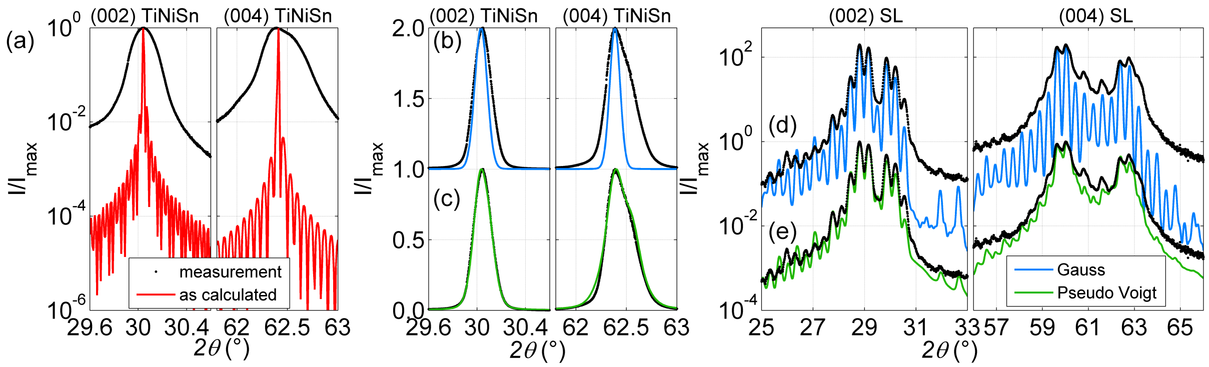

FIG. 1. The calculation (red, blue and green lines) of (002) and (004) reflections for (a-c) TiNiSn and (d-e) $(\mathrm{TiNiSn}_{21.5\,\mathrm{uc}}/\mathrm{HfNiSn}_{21.5\,\mathrm{uc}})\times 29$ SL. The data were convoluted using different peak shapes, which were indicated in the legend. The calculated patterns are compared to the experimental data, displayed in black. Curves were shifted vertically for clarity.

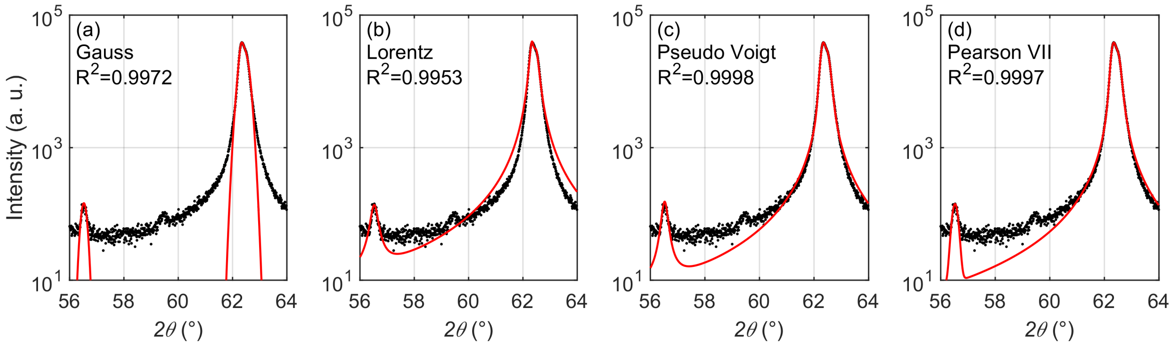

FIG. 2. The comparison of the peak fits (red lines) to the measured (004) TiNiSn reflexion (black data points). Every red line is composed of a triplet with the labeled shape of the peak. The triplet corresponds to the diffraction due to Cu $K_{\alpha 1}$, $K_{\alpha 2}$ and K_{β} radiation lines.

the broadening mechanism proposed by us is reasonable, Figs. 1 and 2 exhibit the XRD patterns acquired from two different 1 μm thick TiNiSn films. As demonstrated in Fig. 1 c one can obtain very good resemblance between two XRD patterns using solely empirical parameters to convolute the peaks. A minor discrepancy between the measured and calculated data of (004) reflection is in our opinion negligible and arises from sample-to-sample scattering of the exact peak shape.

III. CADEM

A. List of files

The source code of CADEM contains the following functions:

- *constants.m* creates a structure that contains general and material dependent constants, such as radiation wavelength, density, lattice constant, mass, mass absorption coefficient, parameters allowing for calculation of the atomic scattering factor, and broadening parameters.
- *atomicScatteringFactor.m* calculates the atomic scattering factor for all elements defined in the function *constants.m*, i.e. V, Ti, Zr, Hf, Ni, Sn, Sr, Ru, and O.
- *choose_system.m* assigns the lattice constants, the atomic scattering factors, and the mass absorption coefficients to the variables used in the *CADEM.m*, depending on the selected system (1 = V, 2 = substrate/TiNiSn/HfNiSn..., 3 = substrate/HfNiSn/TiNiSn..., 4 = $SrRuO_3$, 5 = $SrTiO_3$).

- *CADEM.m* calculates the intensity of diffracted x-rays based on the values assigned in the function *choose_system.m*.

- *run_CADEM.m* calls all functions necessary for the calculation of the intensity of diffracted x-rays. The calculation is performed based on the data saved in the configuration file (its structure is described below), whose path is passed as an argument of the *run_CADEM.m* function. Subsequently the calculated pattern is saved in the text file.

- *run_CADEM_GUI.m* allows to calculate XRD patterns without the necessity of using the configuration file. All necessary parameters can be specified in the graphical user interface. There exist a possibility to load a simulation parameters from an existing configuration file, generate an updated configuration file, and save the calculated data. Moreover, it is also possible to load measured data and display them together with the calculated ones.

There are two ways to perform the calculations:

1. Run a function called *run_CADEM_GUI* to start a graphical user interface.

2. Run a function *run_CADEM(ConfFilePath)*, where *ConfFilePath* is a path to the configuration file. For details about the configuration file see below.

Both of these methods are equivalent.

Moreover, the repository contains 5 examples of configuration files, called *cf1.dat* ... *cf5.dat*, and 2 examples of arbitrary stacks of layers, i.e *ArbitraryStack1.dat* and *ArbitraryStack2.dat*.

B. The structure of the configuration file

The configuration file consists of 17 rows and 2 columns. The fist column contains description of the parameter that is specified in the second column. Detailed description:

1. *system* is equal to one of the following: 1 = V, 2 = substrate/TiNiSn/HfNiSn..., 3 = substrate/HfNiSn/TiNiSn..., 4 = $SrRuO_3$, 5 = $SrTiO_3$.

2. *2theta_002_from* is the starting value of 2θ for the calculation of (002) reflection.

3. *2theta_002_step* is the calculation step for (002) reflection.

4. *2theta_002_to* is the final value of 2θ for the calculation of (002) reflection.

5. *2theta_004_from* is the starting value of 2θ for the calculation of (004) reflection.

6. *2theta_004_step* is the calculation step for (004) reflection.

7. *2theta_004_to* is the final value of 2θ for the calculation of (004) reflection.

8. *layerA_latticeconstant_A* is the lattice constant (in Å) of a layer that lies on top of the substrate. In case of system = 2, layer A is TiNiSn.

9. *layerB_latticeconstant_A* is the lattice constant (in Å) of the layer B. In case of system = 2, layer B is HfNiSn.

10. *periodicArrangement* is an argument that allows to specify if the investigated layer arrangement is periodic (then set value equal to 1) or non-periodic (any other value). Periodic arrangement means a SL with well defined SL period and number of bilayers. Non-periodic arrangement can be any arbitrary layer stack that will be loaded from a file. For details about the file structure see point 14.

11. *layerA_thickness_uc* is the thickness of layer A in unit cells. In case of system = 2, layer A is TiNiSn.

12. *layerB_thickness_uc* is the thickness of layer B in unit cells. In case of system = 2, layer B is HfNiSn.

13. *numberOfbilayers* an integer number of periods (bilayers) of the periodic layer arrangement. E.g. by setting this value to 1 and *layerB_thickness_uc* = 0 one can study finite size oscillations of layer A.

14. *non-periodicArrangement_file* a path to the file that contains non-periodic layer arrangement, e.g. *ArbitraryStack1.dat*. A file can have .txt or .dat extension. It must contain an even number of elements. Assuming system = 2, a series of numbers [13; 14; 2; 8] saved in a file will mean: substrate/$TiNiSn_{13\,uc}$/$HfNiSn_{14\,uc}$/$TiNiSn_{2\,uc}$/$HfNiSn_{8\,uc}$, conversely [13; 14; 2; 0] will skip the last HfNiSn layer, however, it must be specified for correct operation of the program.

15. *numberOfUnitCellsOfIntermixing* is the length (in unit cells) at the interface, where the intermixing takes place. E.g. if intermixing = 2 uc, the lattice parameters and the atomic scattering factors are modified along the length of 1 uc for layer A and 1 uc for layer B. In case of intermixing = 4 uc, the same happens for 2 uc of layer A and 2 uc of layer B.

16. *SingleLayer_latticeconstant_A* is the lattice constant of a single material in unit cells. Must be specified if system = 1, 4, or 5.

17. *SingleLayer_thickness_uc* is the thickness of a single material in unit cells. Must be specified if system = 1, 4, or 5.

The files *cf1.dat* ... *cf5.dat* are few examples of configuration files.

C. How to add a new material

To add a new material go to the file *constants.m* and specify material dependent constants accordingly to the existing structure. On an example of vanadium:

```
% Vanadium
const.a.V = [10.2971 7.3511  2.0703   2.0571];  % see Eq.(7) in Supplementary Material
const.b.V = [ 6.8657 0.4385 26.8938 102.478];   % see Eq.(7) in Supplementary Material
const.c.V = [ 1.2199 0       0        0];       % see Eq.(7) in Supplementary Material
const.B.V = 0.5855;                             % Debye-Waller factor in A^2
const.disp_corr_1.V = 0.2;                      % Delta f'  - see Eq.(8) in Supplementary Material
const.disp_corr_2.V = 2.3;                      % Delta f'' - see Eq.(8) in Supplementary Material
const.d.V           = 3.02;                     % lattice constant in angstroem
const.density.V     = 6.11;                     % density in g/cm^3
const.masAbsCoeff_alpha.V  = 222.6;             % Mass attenuation coefficient mu/rho (cm^2/g)
const.masAbsCoeff_beta.V   = 168.0;             % see Eq.(10) in Supplementary Material
                                                % values for Cu Kalpha and Cu Kbeta
const.mass.V     = 50.94;                       % mass (g/mol)
```

Moreover, it is necessary to determine how many atomic planes form a single unit cell. In case of *M*NiSn one can distinguish 4 atomic layers, i.e. *M*Sn/Ni/*M*Sn/Ni. On the other hand, in case of vanadium, or any material crystallizing in fcc or bcc structure, there are only 2 layers. In each of them there is only one atom. Therefore, one must specify a constant `const.divide_uc` that stores this information. If an exemplary material would be composed of 5 uc and it would be saved as e.g. system = 6, one should create another case (in the file *constants.m*):

```
switch system
    case {1, 4, 5}
        const.divide_uc = 2;        % e.g. V has bcc unit cell and can be divided into 2 layers
    case {2, 3}
        const.divide_uc = 4;        % HH materials can be divided into 4 atomic layers
    case 6
        const.divide_uc = 5;        % an exemplary material that is composed of 5 unit cells
end
```

Then go to the file *choose_system.m* and define a new system you want to investigate. If you would like to investigate an exemplary material that has 5 atomic layers in a unit cell, create a new system. Its number must be equal to a value that is not in a use yet (e.g. system = 6). For vanadium system = 1:

```
switch system
    case 1 % Vanadium
    f.Ka1.a1 = f.Ka1.V;  % assign atomic scattering factor to a variable f.wavelength.a1
```

```
    f.Ka2.a1 = f.Ka2.V;  % that is used in the function CADEM as a first layer of the unit cell
    f.Kb.a1  = f.Kb.V;
    f.Ka1.a2 = f.Ka1.V;  % assign atomic scattering factor to a variable f.wavelength.a2
    f.Ka2.a2 = f.Ka2.V;  % that is used in the function CADEM as a second layer of the unit cell
    f.Kb.a2  = f.Kb.V;

    if isnan(const.d_a_new)     % if you do not specify a new value of lattice constant
                                % (const.d_a_new),
        const.d_a = const.d.V/const.divide_uc;       % the bulk value will be used
    else                                             % otherwise,
        const.d_a = const.d_a_new/const.divide_uc;   % a value specified by a user will be used
    end

    % calculate the linear attenuation coefficient for Cu Kalpha and Kbeta radiation wavelengths
    const.mu_Ka = const.masAbsCoeff_alpha.V * const.density.V; % (1/cm)
    const.mu_Ka = const.mu_Ka/0.01;                            % (1/m)

    const.mu_Kb = const.masAbsCoeff_beta.V * const.density.V; % (1/cm)
    const.mu_Kb = const.mu_Kb/0.01;                           % (1/m)
end
```

For more complex materials, like e.g. half-Heusler TiNiSn it is necessary to calculate atomic scattering factors and attenuation coefficients based on a percentage share of elements in a single atomic layer. For details we refer to the source code and Eq. (11)–(12).

[1] M. J. Buerger and G. E. Klein, J. Appl. Phys. **16**, 408 (1945).

[2] C. H. MacGillavry, G. D. Rieck, and K. Lonsdale, eds., *International Tables for X-Ray Crystallography, Volume III: Physical and Chemical Tables* (Kynoch Press, Birmingham, 1968).

[3] J. A. Ibers and W. C. Hamilton, eds., *International Tables for X-Ray Crystallography, Volume IV: Revised and Supplementary Tables* (Kynoch Press, Birmingham, 1974).

[4] H. Templeton, Acta Cryst. **8**, 841 (1955).

[5] L. Peng, G. Ren, S. L. Dudarev, and M. J.Whelan, Acta Cryst. **A52**, 956 (1996).

[6] M. Birkholz, P. F. Fewster, and C. Genzel, *Thin film analysis by x-ray scattering*, edited by M. Birkholz (Wiley-VCH, Weinheim, 2006).

[7] L. V. Azároff, Acta Cryst. **8**, 701 (1955).

[8] A. J. Ying, C. E. Murray, and I. C. Noyan, J. Appl. Crystallogr. **42**, 401 (2009).

[9] G. Gładyszewski, Thin Solid Films **170**, 99 (1989).

[10] G. Gładyszewski, Thin Solid Films **204**, 473 (1991).
